# Development of quantitative and concise measurement method of oxygen in fine bubble dispersion

**DOI:** 10.1371/journal.pone.0264083

**Published:** 2022-02-16

**Authors:** Kenta Kakiuchi, Takehiro Miyasaka, Norikazu Harii, Shinji Takeoka

**Affiliations:** 1 Faculty of Science and Engineering, Waseda University (TWIns), Shinjuku, Tokyo, Japan; 2 Department of Materials and Human Environmental Science, Shonan Institute of Technology, Fujisawa, Kanagawa, Japan; 3 Department of Community and Family Medicine, Faculty of Medicine, University of Yamanashi, Chou, Yamanashi, Japan; 4 Institute for Advanced Research of Biosystem Dynamics, Research Institute for Science and Engineering, Waseda University, Shinjuku, Tokyo, Japan; University of North Carolina at Chapel Hill, UNITED STATES

## Abstract

Fine bubbles (FBs) have attracted significant attention in several research fields. Although some reports have argued that FB dispersion is useful as an oxygen (gas) carrier, only a few reports have examined its properties as an oxygen carrier using experimental data. As one of the reasons for this, there are no standard methods for measuring the oxygen content in FB dispersions. Conventional oxygen measurement methods have certain drawbacks in accuracy or speed; thus, it is difficult to use oxygen content as the primary outcome. In this study, we introduce a Clark-type polarographic oxygen electrode device (OXYG1-PLUS) for oxygen measurement, allowing the dilution of FB dispersion without the influence of ambient air and the adhesion of FBs on the electrode surface due to its special shape. First, the accuracy of our dilution method was evaluated using pure water as a sample, and it was confirmed that our method could measure with an accuracy of ±0.5 mg/L from the results with conventional dissolved oxygen meters. Second, the oxygen content in FB dispersion was evaluated with our method and a chemical titration method (Winkler’s method), and it was found that our method could measure the oxygen content in FB dispersions quantitively. This method satisfies the easiness (4 steps) and quickness (within 8 min) for a wide range of oxygen contents (0 to 332 mg/L, theoretical range) with low coefficient variation (< 4.7%) and requires a small sample volume (50–500 μL); thus, it is a useful method for measuring the oxygen in FB dispersions.

## Introduction

Fine bubbles (FBs) are tiny floating bubbles that were internationally standardized in 2013 (ISO/TC 281) and consist of microbubbles (MBs) with diameters ranging from 1 to 100 μm and ultrafine bubbles (UFBs, also known as bulk nanobubbles) with diameters of less than 1 μm [1]. FBs have unique properties owing to their small size, such as a long retention time [2, 3], negative surface charge [4, 5], high internal pressure [6, 7], and large specific surface area [6, 8]. In particular, stability of UFBs (100 to 200 nm) in liquid was reported from several researchers, and they claimed that UFBs exist in saturated water for over one month [9–11]. However, the mechanism of the stability is still ongoing issue although some hypotheses have been reported [12–15]. Based on these features, FBs have been used as a gas carrier, particularly as an oxygen carrier, and have been applied in the fishing industry [16, 17], water treatments [18, 19], chemical reactions [20–22], biological applications [17], and medicine [23–25]. Because FB dispersions can be prepared in a large scale and within a short time at a low cost, they are expected to be further developed for various applications. However, few studies have examined the fundamental properties of FBs as oxygen carriers because conventional methods for measuring the oxygen content in FB dispersions have drawbacks in terms of accuracy or quickness. Therefore, a novel method that satisfies the required accuracy, ease, and speed is needed in this field.

In recent studies, dissolved oxygen (DO) meters [2, 9], a blood gas analyzer [24], and a titration method (Winkler’s method) [17, 26] have been used to measure the oxygen content in FB dispersions under the temperature region from 20°C to 30°C. Although the two former methods can provide data quickly and easily, the accuracy is concerned when FBs attach to the surface of their electrodes [27], and it should be noted that the oxygen content in the gas phase of the FBs is not included in the outcome. Moreover, conventional DO meters cannot measure dissolved oxygen concentrations of above 50 mg/L. By contrast, Winkler’s method is known to achieve precise measurement and has a wide measurement range. However, it requires complicated procedures and a time-consuming preparation of the reagent.

Dilution methods were used for measuring the oxygen in stabilized MB dispersions [8, 28]. Lipid-coated MBs were developed to deliver a copious amount of oxygen (50–90 vol%) [28] and can therefore be administered to animals intravenously or intraperitoneally for temporal systemic oxygen delivery [29, 30]. Since conventional DO meters cannot be used for measuring the oxygen content in the oxygen carrier dispersion due to its low concentration range (< 50 mg/L), the oxygen content was evaluated by injecting the specific amount of the sample dispersion into deoxygenated water and assessing the level of oxygen in the mixed water (dilution method). The authors developed a complicated flow system incorporating micro-oxygen electrodes to mix a sample dispersion and deoxygenated water in a closed system [28]. Furthermore, since the sample dispersion was introduced into the system with a syringe pump, the stability for several minutes was required for samples, that is hard for non-coated MBs. Thus, although it was an excellent system it included some limitation in versatility and available samples. Although similar dilution methods with conventional DO meters was reported to measure the oxygen content in polysaccharide-coated FB dispersions, they require a large amount of deoxygenated water (20–120 mL) and samples (3–12 mL) in each measurement [8, 31]. Furthermore, it is difficult for these methods to eliminate the influence of ambient air because they used general beakers and stirring system under a condition that was not completely closed, and they did not conduct quantitative evaluation of oxygen content.

We have tried to improve the dilution methods in terms of the influence of oxygen in ambient air and bubble attachment on the electrode surface with a simple device. Moreover, we believe that the measuring time can be shortened by reducing the amount of deoxygenated water required for a single measurement because deoxygenation from bubbling nitrogen (N_2_) is time consuming. We found that a Clark-type polarographic oxygen electrode device (OXYG1-PLUS; Hansatech Instruments Ltd, Norfolk, UK), which is a simple system used for measuring the liquid-phase photosynthesis and respiration of plants [32], had a suitable structure to achieve the above aims. The purpose of this study is to develop a novel dilution method using this device to quantify the oxygen content in FB dispersions more quickly and easily. To achieve the purpose, the accuracy of our dilution method was evaluated using pure water as a sample by comparing with the results measured with conventional DO meters, and the oxygen content in the FB dispersion was evaluated by comparing the results with those obtained by the Winkler’s method.

## Materials and methods

### Novel method for quantitative oxygen content measurement

**Fig 1:**
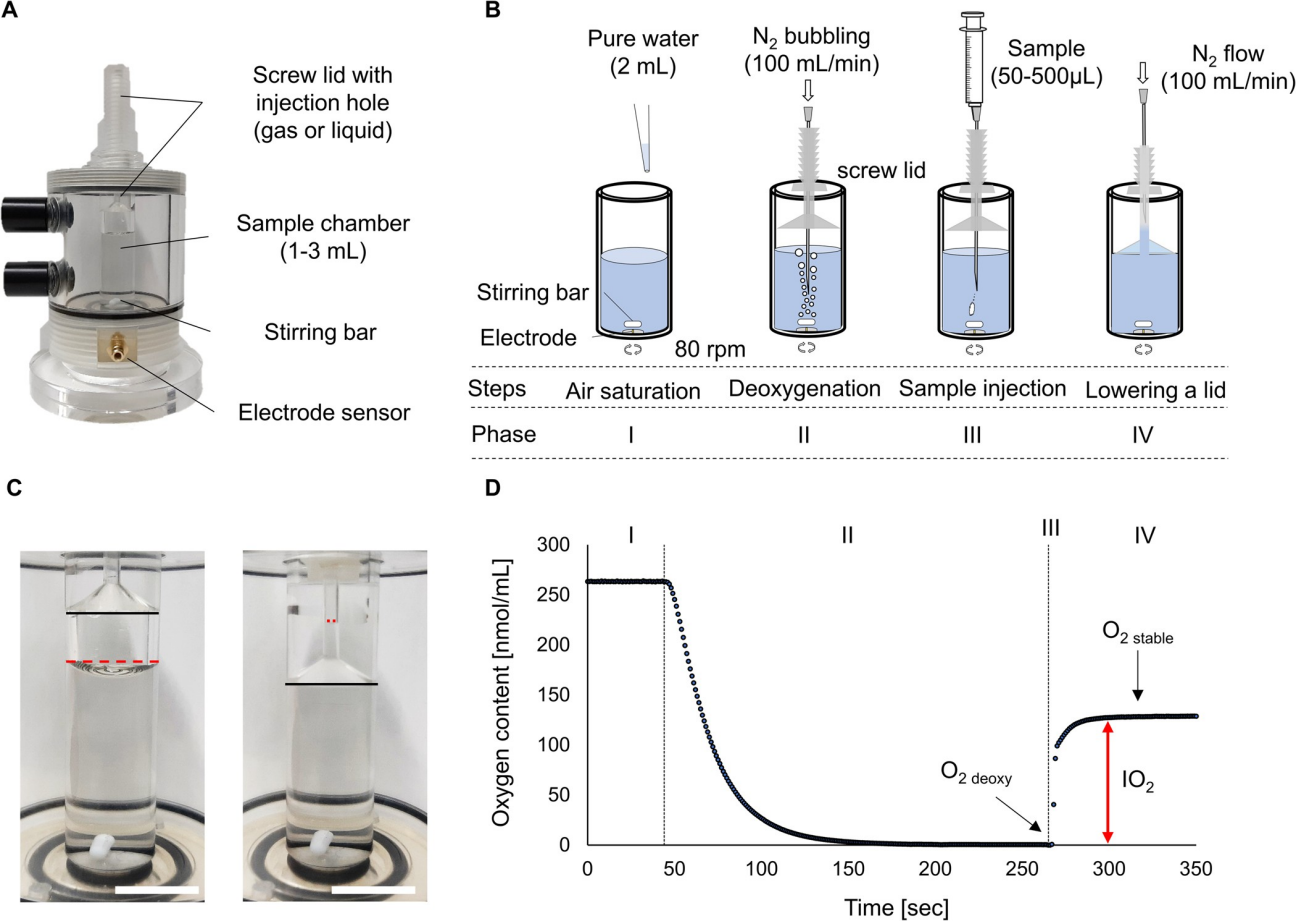
Novel oxygen content measurement for fine bubble dispersions. (A) A Clark-type oxygen electrode. (B) Schematic illustration of measurement procedures, which consist of four steps: air saturation (phase I), deoxygenation (phase II), sample injection (phase III), and lowering a lid (phase IV). (C) Change in the air-liquid interface using a screw lid. Black lines, the bottom of the screw lid; discontinuous red line, surface of pure water. Scale bar, 10 mm. (D) Change in oxygen content during a measurement. A two-way arrow indicates the level of oxygen increment (IO_2_) with a sample injection.

OXYG1-PLUS has a screw lid that can control the contact of the gas–liquid interface in the sample chamber (Fig 1A). Since an electrode disk is placed at the bottom of the device (S1 Protocol and S1 Fig), and a stirring bar rotates on the electrode surface, FBs are hard to adhere to the electrode. The novel method applied in this study consisted of four steps, as shown in Fig 1B. I) Pure water (2 mL) was introduced into the sample chamber and stirred at 80 rpm at room temperature. II) The water was then deoxygenated by bubbling N_2_ (100 mL/min) for 2 to 3 min using a 70-mm needle (22G Cathelin needle; Terumo Corporation, Tokyo, Japan). III) A sample FB dispersion (50 to 500 μL) was injected using a microsyringe with a 70-mm needle after confirming that the oxygen concentration in the pure water was less than 1.0 nmol/mL (O_2 deoxy_). In this method, MBs do not float and disappear in the microsyringe from the sampling to the measurement. IV) Immediately after a sample injection, the screw lid was lowered just at the position of the pure water surface to minimize contact with ambient air (Fig 1C). In addition, N_2_ gas was flowed over the small water surface in the 1-mm hole of the screw lid to eliminate the contamination of oxygen from ambient air, and it was confirmed that the N_2_ gas did not affect the oxygen level in the chamber due to the slight gap at the gas-liquid interface (Fig 1D). This screw lid is important for accurate measurement without effect of ambient gas (S2 Fig). All procedures were performed at room temperature (20–25°C). The temperature of the measurement environment and deoxygenated water has little effect if calibration is performed under the temperature. The oxygen level in the pure water after sample injection (sample diluent) increased only because of the addition of oxygen originated from the injected sample, and it became stable within 60 sec (Phase IV in Fig 1D). Changes in the oxygen content of the sample diluent were monitored and recorded using a software (OxyTrace+; Hansatech Instruments Ltd, Norfolk, UK), as shown in Fig 1D. The measurement was deemed complete when the oxygen level became constant (O_2 stable_). The oxygen content in the sample was calculated from the level of oxygen increment (*IO*_*2*_, nmol/mL) and sample volume (*V*_*s*_, mL), as shown in Eq (1). To compare the results of the DO meters, the unit of oxygen content in the sample was converted into milligrams per liter from nanomoles per milliliter:

$$oxygen\;content\;[mg/L]=\frac{IO_{2}\times (2+V_{S})}{V_{S}}\times \frac{32}{1000}$$ (1)

$${IO}_{2}\;[nmol/mL]=O_{2\;stable}-O_{2\;deoxy}$$

where *IO*_*2*_ is the level of oxygen increment (nmol/mL), and 2+*V*_*S*_ is the total liquid volume in a chamber: deoxygenated pure (2 mL) water and sample volume (*V*_*S*_).

### Accuracy of the oxygen content measurement

First, the coefficients of variation (*CV*, %) for the novel method were examined for all combinations of five sample volumes (50, 100, 300, 450, and 500 μL), and four different temperatures (10°C, 20°C, 30°C, and 40°C) of pure water as sample, with 20 conditions in total. *CV* is a parameter of the relative standard deviation and is calculated using Eq (2):

$$CV\;[\%]=\frac{\sigma }{\mu }\times 100$$ (2)

where *σ* is the standard deviation, and *μ* is the mean.

Air-saturated pure water (3 L) was prepared by bubbling air with stirring (300 rpm) for more than 30 min. The temperature was controlled using a thermostat (ZS-211; ZENSUI Co., Ltd., Osaka, Japan) connected to a heater (NISSO protect PRO heater; Marukan Co., Ltd., Osaka, Japan) and a magnetic pump (MD-6K-N; IWAKI Co., Ltd., Tokyo, Japan) for circulating cold water in a cooling coil. The system can control the temperature of the water within a target temperature of ± 1°C. As a control experiment of the novel method, a DO meter (DO meter 1; OM-71, HORIBA, Ltd., Kyoto, Japan) was immersed in air-saturated water and the oxygen content was measured just before the sampling.

Second, the measurement accuracy was evaluated with pure water at four temperatures (10°C, 20°C, 30°C, and 40°C) as sample by comparing the results obtained through measurements with three different DO meters (DO meter 1; DO meter 2; HI9146, HANNA Instruments, Inc., Rhode Island, USA, and DO meter 3; Seven2Go Pro, Mettler Toledo, Ohio, USA) and a standard oxygen level in an air-saturated water (JIS0120: Japanese industry standard). Air-saturated pure water was used as the sample and prepared in the same way as described above. DO meters 1 and 2 are immersion probe-type DO sensors and adopt electrochemical determinations, *i*.*e*., galvanometric and polarographic methods, respectively. These DO meters detect current that varies according to the oxygen molecules that permeate a diaphragm attached to the tip of the probe. DO meter 3 is also an immersion probe-type DO sensor that adopts fluorometric determination. The determination uses a phenomenon in which a fluorescence excited by the light of a blue light-emitting diode (LED) is quenched by oxygen molecules. The DO was calculated from the degree of fluorescence quenching by oxygen molecules that permeated the diaphragm attached to the tip of the probe.

### Oxygen content in oxygen fine bubble dispersions

The oxygen contents in the FB dispersions at four temperatures (15°C, 20°C, 30°C, and 40°C) were measured using the novel method and three DO meters (DO meter 1, 2 and 3). Since FB dispersions are expected to be used in various applications, samples were prepared in a wide range of temperatures. The lowest temperature was set at 15°C because it is difficult to control the temperature below 15°C due to the heat generated by the FB generator, and the highest temperature was set at 40°C in consideration of the use in biological and medical research (36–38°C). The sample volumes of the FB dispersions were within 100 to 300 μL; hence the dilution rate ranged from 7 to 21-time dilution (detail in S1 Dataset). The FB dispersions were generated in 5 L of pure water in an 8 L of plastic container using a FB generator (Ultrafine GALF FZ1N-02; IDEC Corporation, Osaka, Japan), which adopts a pressurized dissolution method, for more than 15 min under the following conditions: oxygen supply of 0.5 L/min, dissolution pressure of 270–320 kPa, and pump pressure of 1390–1420 kPa. The temperature of the liquid was controlled using the same system as the experiment described in previous subsection (***Accuracy of the oxygen content measurement)***. For the FB dispersion at 15°C, 20°C, and 30°C, to examine the accuracy of the novel method, the oxygen content was measured using Winkler’s method (S2 Protocol). Next, the oxygen content in the FB dispersion was compared to a theoretical DO in oxygen-saturated water calculated from the standard DO in air-saturated water, corrected based on the oxygen ratio of 21% to 100%.

Size distribution and concentration of FBs at 20, 30, and 40°C were evaluated by an NTA instrument (NanoSight LM10, Malvern Panalytical, Malvern, U.K.) for UFBs and a particle size analyzer (PartAn SI, Microtrac-bel Corp., Osaka, Japan) for MBs.

## Results and discussion

### Accuracy of oxygen content measurement

**Fig 2:**
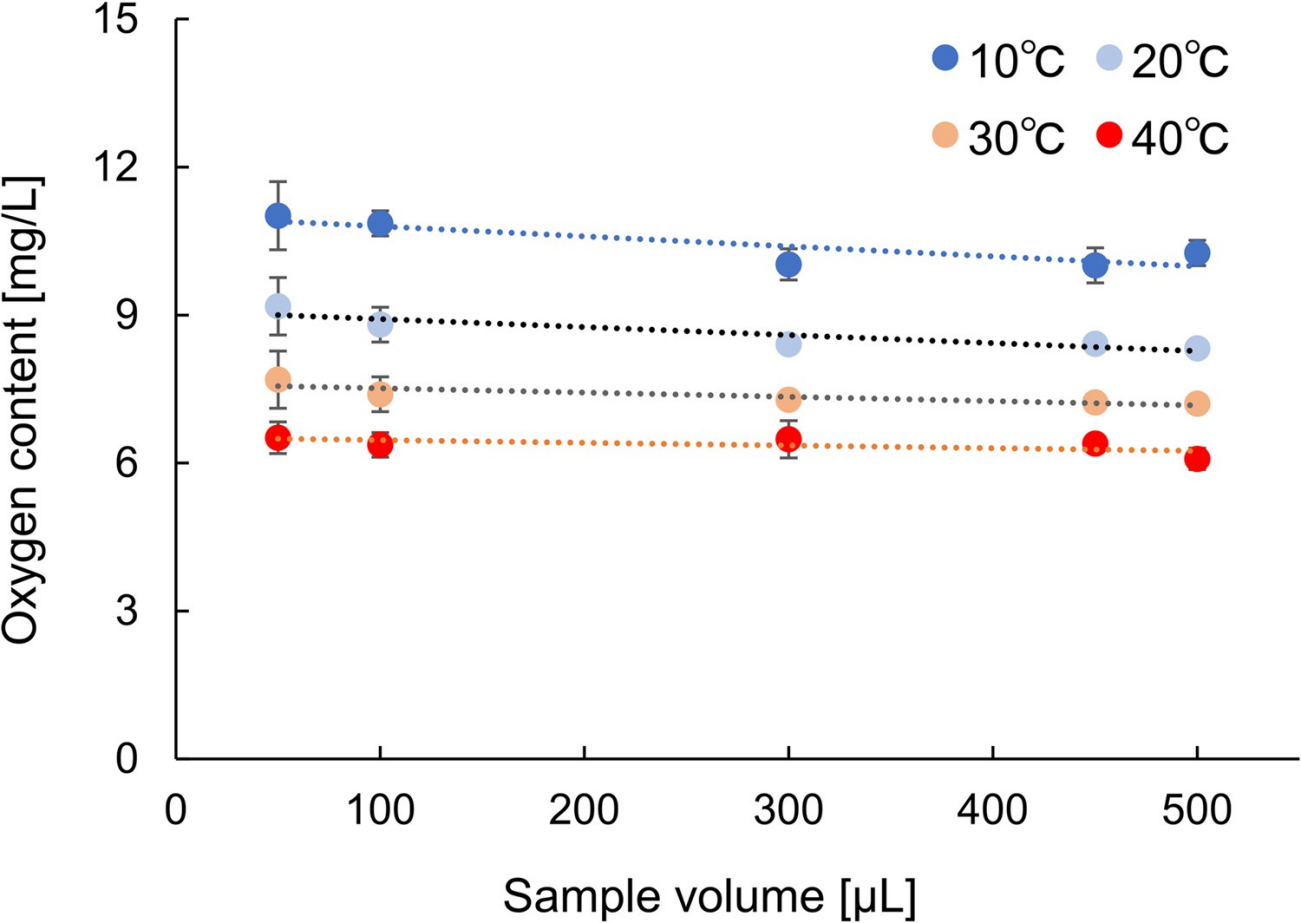
Relationship between oxygen content and sample volume at different temperatures. DO in air-saturated water at 10°C (blue, n = 5), 20°C (light blue, n = 5), 30°C (orange, n = 5), and 40°C (red, n = 5). Data are presented as the mean ± standard deviation.

Fig 2 shows the relationship between the DO and sample volume at four temperatures. The DO values and standard deviations (SDs) tended to become higher and larger, respectively, for smaller sample volumes. Since the result for *IO*_*2*_, *i*.*e*., a parameter prior to the correction, showed a strong correlation with the sample volume (S1 Fig, r > 0.99) and no large variations were confirmed at small sample volumes (S1 Table), the correction based on the sample volume in the calculation process of the oxygen content (Eq (1)) induced this tendency. Table 1A shows the *CV* for each sample volume under all temperature conditions and shows that relatively large *CV*s are shown in small sample volumes (50 and 100 μL). Because the SDs of *IO*_*2*_ were not large in the small sample volumes, the large *CV* was induced by small values of *IO*_*2*_ (S1 Table). This means that sufficient *IO*_*2*_ is necessary to ensure a high accuracy. When the sample volume ranged from 450 to 500 μL, the *CV*s were almost the same as those of DO meter 1; thus, more than 36 nmol/mL of *IO*_*2*_ is preferable to measure with a low *CV* (S1 Table: 450 μL at 40°C). Table 1B shows the *CV*s at different temperatures for all sample volumes. This indicates that the influence of temperature on the *CV* was smaller than that of the sample volume; a sample at 10 to 40°C could be measured with a *CV* of 4% or less. Moreover, if the results obtained with a 50-μL sample volume were excluded, the average *CV* improved from 2.8% to 3.2% (S2 Table).

**Table 1:** Coefficient of variation in a novel oxygen content measurement.

A			B		
	Coefficient of variation [%]		Coefficient of variation [%]		
Volume	Novel method	DO meter 1	Temp.	Novel method	DO meter 1
50 μL	4.7±2.1	2.3±0.6	10°C	3.6±1.4	2.5±0.4
100 μL	3.6±0.7	2.1±0.7	20°C	3.0±1.9	2.5±0.6
300 μL	3.0±1.8	2.2±0.6	30°C	2.2±1.0	1.9±0.5
450 μL	2.2±0.9	2.1±0.2	40°C	3.9±1.5	2.2±0.3
500 μL	2.5±0.7	2.6±0.3	**Ave.**	**3.2**±**0.6**	**2.3**±**0.3**
**Ave.**	**3.2**±**0.9**	**2.3**±**0.2**			

Coefficient of variation for different (A) sample volumes (n = 4 in each volume) and (B) sample temperatures (n = 5 at each temperature). Data are presented as the mean ± standard deviation. DO, dissolve oxygen; Temp., temperature; Ave., average.

In summary, sufficient *IO*_*2*_ (> 36 nmol/mL) is important for reducing the variation of outcomes when using the novel method, and the effect of temperature on *CV* is small. With this device, a calibration of the electrode is applied by adjusting the oxygen level in air-saturated water, which is 253.4 nmol/mL (8.11 mg/L, 25°C). Hence, an accurate measurement can be achieved by adjusting the sample volume such that *IO*_*2*_ is placed between 36 and 253 nmol/mL; the optimal sample volume is determined by the amount of oxygen in the sample.

**Fig 3:**
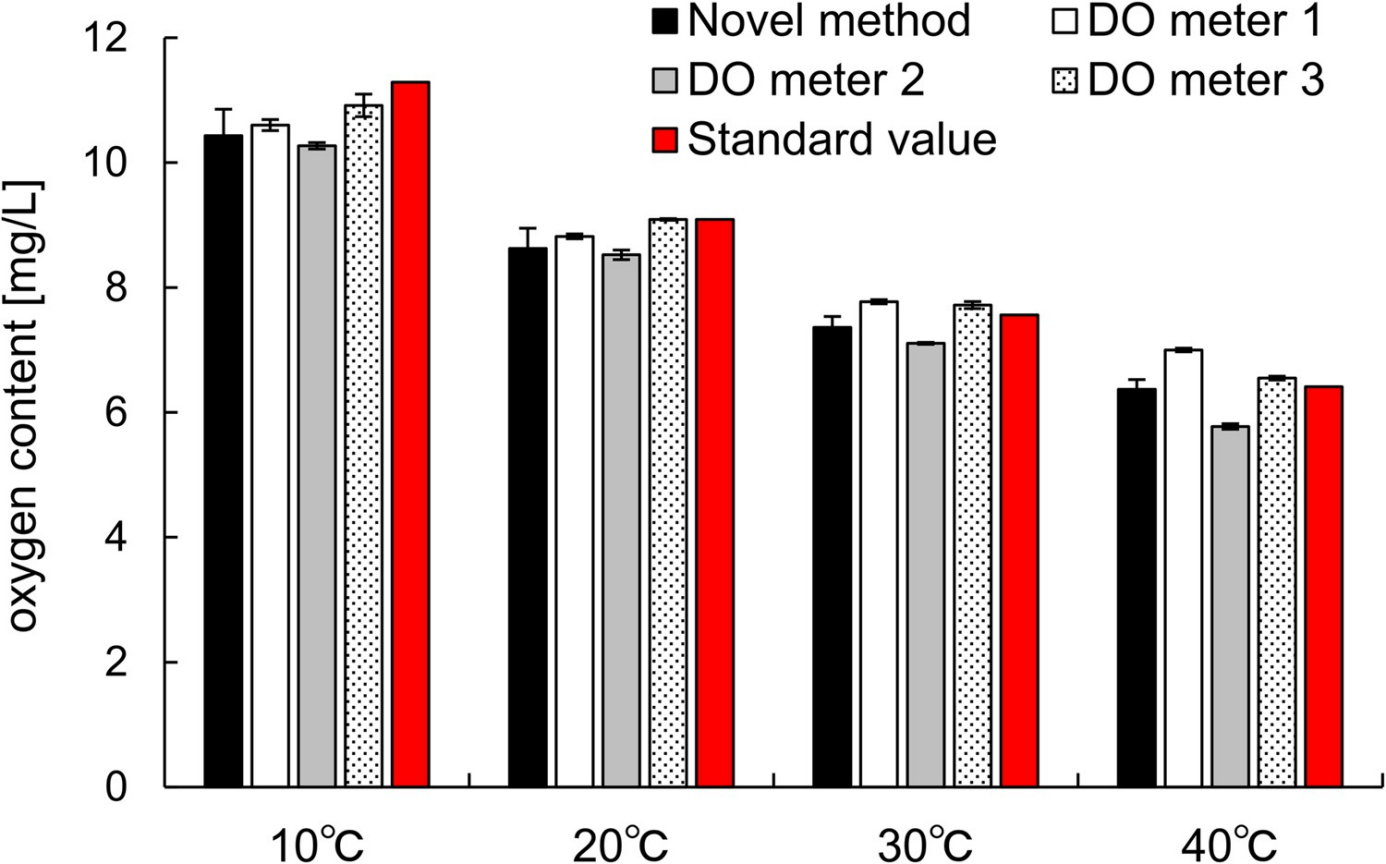
Comparison of accuracy of a novel oxygen content measurement using conventional dissolved oxygen meters. DO in air-saturated water measured using the novel method (black bars, n = 5), DO meter 1 (white bars, n = 5), DO meter 2 (gray bars, n = 5), and DO meter 3 (dot bars, n = 5). Red bars represent standard values (Japanese industry standard 0120). Data are presented as the mean ± standard deviation.

Next, we discuss the accuracy of the measured values by comparing the results obtained using conventional DO meters. Fig 3 shows the oxygen content in air-saturated water at four temperatures measured using the novel method, three different DO meters, and the standard values (JIS0120). Oxygen content in pure water decreased with increasing temperature in all measurement method; it is a well know phenomenon of dissolved oxygen [33]. All values at 10°C were lower than the standard. It is possible that water at 10°C did not reach an air-saturated state of 10°C with contacting room temperature atmosphere. However, the values of the novel method were within those of the DO meters, that is, they were within a difference of 0.5 mg/L from the those of the DO meters, except at 40°C for DO meters 1 and 2, which were far from the standard value. DO meter 1 and 2 tended to show higher and lower values than the other methods, respectively, and this tendency was clearly confirmed at 40°C.

**Fig 4:**
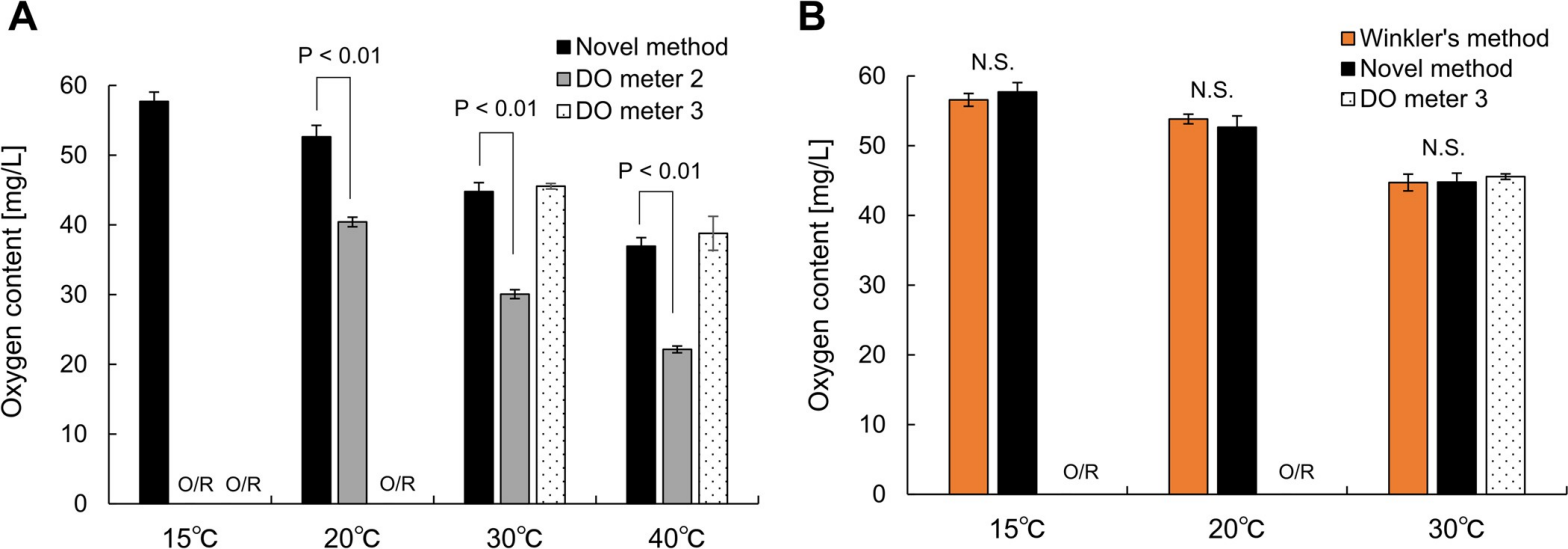
Oxygen content in fine bubble dispersions. (A) Oxygen content in fine bubble dispersion at several temperatures measured using the novel method (black bars, n = 5), DO meter 2 (gray bars, n = 5), and DO meter 3 (dotted bars, n = 5). (B) Oxygen content in fine bubble dispersion measured using Winkler’s method (orange bars, n = 5), the novel method (black bars, n = 5), and DO meter 3 (dotted bars, n = 5). Data are presented as the mean ± standard deviation. Comparisons between the results of novel method at each temperature were examined with the student t test. O/R indicates over the range. N.S. means not significant.

The results in Fig 2 suggest that the value of 50 μL was larger than those of 100–500 μL. Because the resolution of the Clark-type polarographic oxygen electrode is 0.0596 mg/L, a 50 μL volume with a 20-fold correction could induce an error of up to 1.2 mg/L. Although the error has an impact on the oxygen content in pure water, it has a small effect on samples with a high oxygen content (Fig 4).

### Oxygen contents in oxygen fine bubble dispersions

We confirmed that the prepared FB dispersion contained UFBs and MBs (S3 Fig). Since the FB dispersion was injected into sample chamber within 10 sec after sampling, the disappearance of MBs due to floating was negligible in the novel method. Fig 4A shows the oxygen contents in oxygen FB dispersions at different temperatures using the novel method or two DO meters (DO meter 2 and DO meter 3). The oxygen content in the FB dispersions decreased with increasing temperature regardless of the measurement device. It is because that solubility of oxygen in water is inversely proportional to the temperature in liquid [33]. DO meter 1 was unable to measure the oxygen contents of all samples owing to its small range of oxygen content (0–20 mg/L). Black bars represent the values obtained using the novel method, and as indicated in the figure, only this method can measure the oxygen content in all samples. The gray bars represent the values obtained using DO meter 2, which did not provide a value at 15°C owing to out of its measurement range. Moreover, the other values at 20°C to 40°C were clearly different from those obtained using the other two methods. It is thought that FBs attached to the electrode surface and induced the incorrect values. The value was altered by shaking or tapping the electrode to remove the FBs. Dotted bars represent the values obtained using DO meter 3, which adopts a fluorescence method, and the values are almost the same as those obtained from the novel method. However, DO meter 3 could not measure the oxygen content at 15°C and 20°C because the oxygen content was beyond the measurement range (0 to 50 mg/L). The values given by DO meters 2 and 3 were unstable during the measurement; thus, we needed to read the displayed values instantaneously. Furthermore, in the case of DO meter 3, it was also confirmed that the value tended to decrease when the bubbles adhered to the sensor surface. Fig 4A shows that DO meter 3 could measure the oxygen content in the FB dispersions; however, it was necessary to pay attention to the position and orientation of the sensor for avoiding the bubble adhesion to the sensor surface [27]. Therefore, it is difficult to directly measure the oxygen content in FB dispersions with conventional DO meters, particularly in areas where MBs and macro bubbles are continuously generated. Although conventional DO meters can be used for the dilution method, the novel method is preferable for a quantitative measurement of the oxygen content in FB dispersions in terms of 1) an adjustable lid removing the influence of ambient air, 2) an electrode placed at the bottom of the chamber, *i*.*e*., FBs do not attach to its surface, and 3) a low required sample volume (100–200 μL). Furthermore, the *CV* of the results of FB dispersions measured by the novel method was 2.6±0.9% (S3 Table), which is almost the same as the *CV*s of conventional DO meters for pure water.

To obtain the most accurate values, we applied Winkler’s method to the FB dispersions at 15°C, 20°C, and 30°C. Fig 4B shows a comparison of the oxygen contents in FB dispersions measured using Winkler’s method, the novel method, and DO meter 3. There were no significant differences in the obtained values. Although the approach is reliable, it took more than 30 min for Winkler’s method to measure the oxygen content of one sample [34]. Because this method uses chemical fixation, the entire oxygen content in the FB dispersion is measured. By contrast, the novel method measures the oxygen content by diluting the sample. Because the sample volume (100–200 μL) is remarkably less than the 2 mL of deoxygenated water (less than 1/10), most of the oxygen in the sample is expected to dissolve into the deoxygenated water. Therefore, this novel method can be used to accurately measure the oxygen content in FB dispersions. Furthermore, the novel method is preferable to Winkler’s method in terms of ease, speed, and required sample volume.

**Fig 5:**
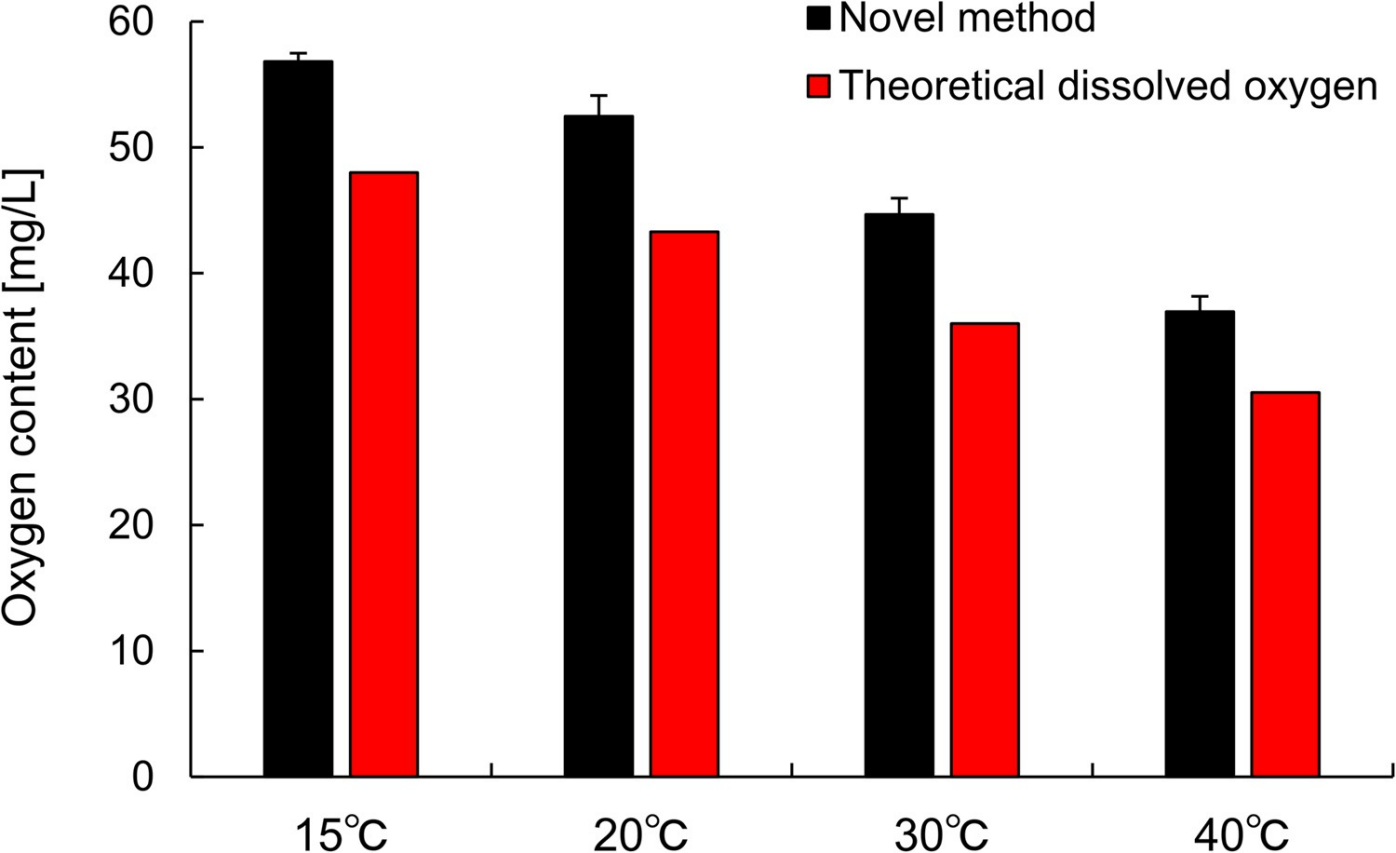
Comparison of oxygen content between FB dispersion and oxygen-saturated water. Oxygen content in fine bubble dispersion at several temperatures measured using the novel method (black bars, n = 5), and theoretical DO in oxygen-saturated water calculated form standard DO in air-saturated water (red bars).

Fig 5 shows the oxygen content in FB dispersions and the theoretical DO in oxygen-saturated water. The FB dispersion had 21±2.4% higher oxygen content than that of oxygen-saturated water, regardless of temperature (S4 Table). There have been a few reports which mentioned the level of oxygen super-saturation in oxygen FB dispersions quantitively, although it was reported on air-saturated FB dispersions which evaluated using DO meters [35, 36]. We have currently been evaluating the relationship between oxygen FB concentration and oxygen content using this method to discuss the effects of FBs on the 21% increment and will report in the next paper. Since this method is applied by taking a 150- to 200-μL sample with a micro-syringe, multiple evaluations can be carried out without any influence on the FBs. Furthermore, it is possible to collect a sample solution from a closed flow channel or cell culture dish if a syringe needle can be inserted [37], and this performance is considered to be difficult to conduct using a conventional DO meter [13]. In this research, shape of the electrode system is important; thus, it is possible to develop a similar system with fluorescence oxygen sensor, not polarographic oxygen electrode, and it is expected to show advantages from the viewpoint of measurement range, applicable solution types, and no stirring is required.

Regarding the measurement range, we experimentally confirmed the novel method could measure the range of 6 to 57 mg/L. However, considering the measurement range of the electrode device itself (OXYG1-PLUS, 0 to 100% oxygen), the theoretical range is expected wider. The oxygen content in air-saturated water at 25°C was 253.4 nmol/mL (8.11 ppm, 21% O2), and if a 50-μL sample increases the oxygen level of 2-mL deoxygenated water from 0 to 253.4 nmol/mL, the sample will contain 332 mg/L of oxygen. Thus, a measurement of 332 mg/L is possible, which is 6.6-times higher than the limitation of conventional DO meters. Although we have to conduct further examination to prove the actual measurement range, we could measure higher oxygen content than that of the conventional probe-type oxygen electrode.

There is a limitation on the experiment. If a sampled dispersion contains macro bubbles (mm scale) with FBs, the accurate value may not be obtained because the macro bubbles rise and disappears while adsorbing FBs. Under conditions where macro bubbles are present, taking care will be required when sampling.

## Conclusion

This novel method can be used to quantitatively measure the oxygen content in FB dispersions. The proposed method is easy (requiring four steps) and rapid (taking <8 min). The measurement range is also wide (0–332 mg/L, theoretical range), and very low sample volumes (50–500 μL) are required. Moreover, the measurement can be conducted using one commercially available device (OXYG1-PLUS); thus, it can be easily introduced in any studies. Therefore, this method is superior to conventional methods for measuring the oxygen content in FB dispersions.

## Supporting information

S1 Protocol OXYG1-PLUS. A summary of the electrode structure and calibration method.
(DOCX)

S2 Protocol Winkler’s method. A summary of how to perform the Winkler’s method we did.
(DOCX)

S1 Fig Structure of electrode disk. (PDF)

S2 Fig Effect of a screw lid on oxygen profile. To evaluate the effect of gas-liquid interface on the oxygen level after injecting sample, the screw lid was lifted again (step V) while monitoring the oxygen content in the liquid. The experiments were conducted under two conditions: the gas phase was nitrogen (A to C) and air (D to F). (A and D) Schematic illustration of measurement procedures, which consist of five steps: air saturation (phase I), deoxygenation (phase II), sample injection (phase III), lowering a lid (phase IV), and lifting a lid (phase V). (B and E) Change in oxygen content during a measurement. Discontinuous red lines indicate time point of event: N_2_ bubbling, sample injection and lowering a lid, and lifting a lid. (C and F) Enlarged view of changes in oxygen content before and after the lifting a lid.
(PDF)

S3 Fig Properties of fine bubble dispersion. Size distribution and concentration of UFBs at A) 20°C, B) 30°C, C) 40°C (n = 3). Approximately 1.0×10^8^ particles/mL were confirmed as same as a manufacturer’s specifications. Size distribution and concentration of MBs at D) 20°C, E) 30°C, F) 40°C (n = 3 at 30°C and 40°C, n = 2 at 20°C).
(PDF)

S4 Fig Relationship between level of oxygen increment and sample volume. Oxygen content in deoxygenated pure water increased with an increase in the sample volume. There was a strong correlation between the two factors under each temperature condition: r = 0.9994 at 10°C, r = 0.9989 at 20°C, r = 0.9988 at 30°C, and r = 0.9960 at 40°C. Data are presented as the mean ± standard deviation (n = 5).
(PDF)

S1 Table Effects of sample volume and temperature on level of oxygen increment. Data are presented as the mean ± standard deviation (n = 5). Ave., average; SD., standard deviation; CV., coefficient of variation.
(PDF)

S2 Table Coefficient of variation without results of a 50-μL sample volume. Data are presented as the mean ± standard deviation (n = 4). Each coefficient of variation includes data under four conditions of sample volume: 100, 300, 450, and 500 μL, excluding 50 μL. Ave., average; SD. standard deviation.
(PDF)

S3 Table Coefficient of variation for results of FB dispersions. Coefficient of variation in each temperature was calculated from the mean and standard deviation in Fig 4A. Ave., average; SD. standard deviation.
(PDF)

S4 Table Oxygen content in FB dispersion and oxygen-saturated water. Mean oxygen content in fine bubble dispersion at several temperatures measured using the novel method (n = 5), and theoretical DO in oxygen-saturated water calculated form standard DO in air-saturated water.
(PDF)

S1 Dataset Measurement data were summarized in an excel file. (XLSX)

## References

[1] Alheshibri M, Qian J, Jehannin M, Craig VSJ. A History of Nanobubbles. Langmuir. 2016;32: 11086–11100. doi: 10.1021/acs.langmuir.6b02489 27594543

[2] Ushikubo FY, Furukawa T, Nakagawa R, Enari M, Makino Y, Kawagoe Y, et al. Evidence of the existence and the stability of nano-bubbles in water. Colloids Surfaces A Physicochem Eng Asp. 2010;361: 31–37. doi: 10.1016/j.colsurfa.2010.03.005

[3] Alheshibri M, Al Baroot A, Shui L, Zhang M. Nanobubbles and nanoparticles. Curr Opin Colloid Interface Sci. 2021;55: 101470. doi: 10.1016/j.cocis.2021.101470

[4] Takahashi M. Zeta potential of microbubbles in aqueous solutions: electrical properties of the gas-water interface. J Phys Chem B. 2005;109: 21858–21864. doi: 10.1021/jp0445270 16853839

[5] Takahashi M, Kawamura T, Yamamoto Y, Ohnari H, Himuro S, Shakutsui H. Effect of shrinking microbubble on gas hydrate formation. J Phys Chem B. 2003;107: 2171–2173. doi: 10.1021/jp022210z

[6] Trasaka K, Himuro S, Ando K, Hata T. Introduction to Fine Bubble Science and Technology (Japanese). The Union of Fine Bubble Scientists and Engineers, editor. NIKKAN KOGYO SHIMBUN,LTD.; 2016.

[7] Oh SH, Kim J. Generation and Stability of Bulk Nanobubbles. Langmuir. 2017;33: 3818–3823. doi: 10.1021/acs.langmuir.7b00510 28368115

[8] Zhao W, Hu X, Duan J, Liu T, Liu M, Dong Y. Oxygen release from nanobubbles adsorbed on hydrophobic particles. Chem Phys Lett. 2014;608: 224–228. doi: 10.1016/j.cplett.2014.05.079

[9] Nirmalkar N, Pacek AW, Barigou M. On the Existence and Stability of Bulk Nanobubbles. Langmuir. 2018;34: 10964–10973. doi: 10.1021/acs.langmuir.8b01163 30179016

[10] Ohmori M, Haruta K, Kamimura S, Koike H, Uchida T, Takeyama H. A Simple Method for Nanobubble Generation and Stability of the Bubbles. J Environ Biotechnol. 2015;15: 41–44.

[11] Jadhav AJ, Barigou M. Bulk Nanobubbles or Not Nanobubbles: That is the Question. Langmuir. 2020;36: 1699–1708. doi: 10.1021/acs.langmuir.9b03532 32040327 PMC7146852

[12] Ghaani MR, Kusalik PG, English NJ. Massive generation of metastable bulk nanobubbles in water by external electric fields. Sci Adv. 2020;6: 1–7. doi: 10.1126/sciadv.aaz0094 32284977 PMC7124953

[13] Ulatowski K, Sobieszuk P, Mróz A, Ciach T. Stability of nanobubbles generated in water using porous membrane system. Chem Eng Process—Process Intensif. 2019;136: 62–71. doi: 10.1016/j.cep.2018.12.010

[14] Nirmalkar N, Pacek AW, Barigou M. Interpreting the interfacial and colloidal stability of bulk nanobubbles. Soft Matter. 2018;14: 9643–9656. doi: 10.1039/c8sm01949e 30457138

[15] Yasui K, Tuziuti T, Kanematsu W, Kato K. Dynamic Equilibrium Model for a Bulk Nanobubble and a Microbubble Partly Covered with Hydrophobic Material. Langmuir. 2016;32: 11101–11110. doi: 10.1021/acs.langmuir.5b04703 26972826

[16] Srithongouthai S, Endo A, Inoue A, Kinoshita K, Yoshioka M, Sato A, et al. Control of dissolved oxygen levels of water in net pens for fish farming by a microscopic bubble generating system. Fish Sci. 2006;72: 485–493. doi: 10.1111/j.1444-2906.2006.01176.x

[17] Ebina K, Shi K, Hirao M, Hashimoto J, Kawato Y, Kaneshiro S, et al. Oxygen and Air Nanobubble Water Solution Promote the Growth of Plants, Fishes, and Mice. PLoS One. 2013;8: 2–8. doi: 10.1371/journal.pone.0065339 23755221 PMC3673973

[18] Agarwal A, Ng WJ, Liu Y. Principle and applications of microbubble and nanobubble technology for water treatment. Chemosphere. 2011;84: 1175–1180. doi: 10.1016/j.chemosphere.2011.05.054 21689840

[19] Khuntia S, Majumder SK, Ghosh P. Microbubble-aided water and wastewater purification: A review. Rev Chem Eng. 2012;28: 191–221. doi: 10.1515/revce-2012-0007

[20] Mase N, Mizumori T, Tatemoto Y. Aerobic copper/TEMPO-catalyzed oxidation of primary alcohols to aldehydes using a microbubble strategy to increase gas concentration in liquid phase reactions. Chem Commun. 2011;47: 2086–2088. doi: 10.1039/c0cc04377j 21203609

[21] Mase N, Nishina Y, Isomura S, Sato K, Narumi T, Watanabe N. Fine-Bubble-Based Strategy for the Palladium-Catalyzed Hydrogenation of Nitro Groups: Measurement of Ultrafine Bubbles in Organic Solvents. Synlett. 2017;28: 2184–2188. doi: 10.1055/s-0036-1588869

[22] Mase N, Isomura S, Toda M, Watanabe N. Micro and nanobubble based strategy for gas-liquid-solid multiphase reactions: Palladium-catalysed hydrogenation of carbon-carbon unsaturated bonds. Synlett. 2013;24: 2225–2228. doi: 10.1055/s-0033-1339798

[23] Matsuoka H, Ebina K, Tanaka H, Hirao M, Iwahashi T, Noguchi T, et al. Administration of oxygen ultra-fine bubbles improves nerve dysfunction in a rat sciatic nerve crush injury model. Int J Mol Sci. 2018;19. doi: 10.3390/ijms19051395 29735961 PMC5983615

[24] Matsuki N, Ichiba S, Ishikawa T, Nagano O, Takeda M, Ujike Y, et al. Blood oxygenation using microbubble suspensions. Eur Biophys J. 2012;41: 571–578. doi: 10.1007/s00249-012-0811-y 22476882

[25] Kakiuchi K, Matsuda K, Harii N, Sou K, Aoki J, Takeoka S. Establishment of a total liquid ventilation system using saline-based oxygen micro/nano-bubble dispersions in rats. J Artif Organs. 2015;18: 220–227. doi: 10.1007/s10047-015-0835-z 25854604

[26] Kikuchi K, Ioka A, Oku T, Tanaka Y, Saihara Y, Ogumi Z. Concentration determination of oxygen nanobubbles in electrolyzed water. J Colloid Interface Sci. 2009;329: 306–309. doi: 10.1016/j.jcis.2008.10.009 18977493

[27] Li Y, Buckin V. State of Oxygen Molecules in Aqueous Supersaturated Solutions. J Phys Chem B. 2019;123: 4025–4043. doi: 10.1021/acs.jpcb.9b01057 30875227

[28] Swanson EJ, Mohan V, Kheir J, Borden MA. Phospholipid-stabilized microbubble foam for injectable oxygen delivery. Langmuir. 2010;26: 15726–15729. doi: 10.1021/la1029432 20873807

[29] Kheir JN, Scharp LA, Borden MA, Swanson EJ, Loxley A, Reese JH, et al. Oxygen gas-filled microparticles provide intravenous oxygen delivery. Sci Transl Med. 2012;4. doi: 10.1126/scitranslmed.3003679 22745438

[30] Feshitan JA, Legband ND, Borden MA, Terry BS. Systemic oxygen delivery by peritoneal perfusion of oxygen microbubbles. Biomaterials. 2014;35: 2600–2606. doi: 10.1016/j.biomaterials.2013.12.070 24439406 PMC7124456

[31] Cavalli R, Bisazza A, Giustetto P, Civra A, Lembo D, Trotta G, et al. Preparation and characterization of dextran nanobubbles for oxygen delivery. Int J Pharm. 2009;381: 160–165. doi: 10.1016/j.ijpharm.2009.07.010 19616610

[32] Gotoh E, Suetsugu N, Yamori W, Ishishita K, Kiyabu R, Fukuda M, et al. Chloroplast accumulation response enhances leaf photosynthesis and plant biomass production. Plant Physiol. 2018;178: 1358–1369. doi: 10.1104/pp.18.00484 30266749 PMC6236601

[33] Benson BB, Krause D. The concentration and isotopic fractionation of oxygen dissolved in freshwater and seawater in equilibrium with the atmosphere. Deep Sea Res Part B Oceanogr Lit Rev. 1984;31: 859. doi: 10.1016/0198-0254(84)93289-8

[34] Ulatowski K, Sobieszuk P. Gas nanobubble dispersions as the important agent in environmental processes–generation methods review. Water Environ J. 2020;34: 772–790. doi: 10.1111/wej.12577

[35] Tanaka S, Terasaka K, Fujioka S. Generation and Long-Term Stability of Ultrafine Bubbles in Water. Chemie-Ingenieur-Technik. 2021. pp. 168–179. doi: 10.1002/cite.202000143

[36] Oshita S, Kamijo Y, Anh PTQ, Yoshimura M, Sotome I, Kameya H, et al. Number concentration of ultrafine bubble being effective in promoting barley seed germination (Japanese). Japanese J Multiph Flow. 2020;34: 194–204. Available: https://www.jstage.jst.go.jp/article/jjmf/34/1/34_2020.020/_pdf

[37] Kakiuchi K, Miyasaka T, Takeoka S, Matsuda K, Harii N. Total alveolar lavage with oxygen fine bubble dispersion directly improves lipopolysaccharide-induced acute respiratory distress syndrome of rats. Sci Rep. 2020;10. doi: 10.1038/s41598-019-56089-4 33024204 PMC7538589

