# Supplementary material for: Development of quantitative and concise measurement method of oxygen in fine bubble dispersion
Source: PLoS One. 2022 Feb 16;17(2):e0264083. doi: 10.1371/journal.pone.0264083 (PMC8849465; doi:10.1371/journal.pone.0264083)
Supplement: S1 Protocol — A summary of the electrode structure and calibration method. (DOCX) [file pone.0264083.s001.docx]

**S1 Protocol. OXYG1-PLUS**

S1A Fig. shows an oxygen electrode disk of the device (OXYG1-PLUS). A body part was made of epoxy resin, and there are two electrodes: a platinum cathode and a silver anode. These two electrodes are connected by a paper containing 50% KCl aq, which functions as an electrolyte (S1B Fig.). The principle of the oxygen electrode is the same as that of general polarographic oxygen electrodes. The electrode section is finally covered with an oxygen-permeable membrane and sealed with an O-ring to separate the electrode section from sample liquid (S1B Fig.).

Liquid-phase calibration was performed with following a manual of the devise. After preparing the electrode disk, the electrode was connected to a control unit with a connection cable and left for several hours for aging. Then, 2 mL of air-saturated pure water was introduced into a measurement chamber (Fig. 1A) and stirred by a magnetic stirrer in the control unit. Air-saturated water was prepared by vigorous shaking in a 50 mL vial. The temperature of the pure water was measured, and calibration was conducted with air-saturated water and deoxygenated water following the calibration system in a software (OxyTrace+; Hansatech Instruments Ltd, Norfolk, UK). Deoxygenated water was prepared with N_2_ bubbling. With this device, deoxygenation can be performed while monitoring the electrical signals; therefore, it is possible to confirm that deoxygenation is performed reliably. Although deoxygenation can be performed with sodium dithionite, nitrogen bubbling is recommended due to the risk of contamination in the measurement chamber and damage to the oxygen permeable membrane.
