# Supplementary material for: Development of quantitative and concise measurement method of oxygen in fine bubble dispersion
Source: PLoS One. 2022 Feb 16;17(2):e0264083. doi: 10.1371/journal.pone.0264083 (PMC8849465; doi:10.1371/journal.pone.0264083)
Supplement: S2 Protocol — A summary of how to perform the Winkler’s method we did. (DOCX) [file pone.0264083.s002.docx]

**S2 Protocol. Winkler’s method**

The chemical reactions of Winkler’s method are shown in S1 Scheme. A Manganese sulfate solution (fixative 1; 2.15 mol/L MnSO_4_aq), an alkali-iodine-azide solution (fixative 2; 6 mol/L NaI in 10 mol/L NaOHaq), a sulfuric acid solution (6.8 mol/L H_2_SO_4_), a 1% starch solution, a sodium thiosulfate solution (0.02 mol/L Na_2_S_2_O_3_), and a potassium iodide solution (0.001667 mol/L KIO_3_) were prepared a few days prior to titration. The exact concentration of the sodium thiosulfate solution was determined immediately before titration by standardization with a potassium iodide solution.

$${Mn}^{2+}+{2OH}^{-}\to Mn\left( OH \right)_{2}\downarrow:White precipitation$$

$$2Mn\left( OH \right)_{2}+{\frac{1}{2}O}_{2}+H_{2}O\to2Mn\left( OH \right)_{3}\downarrow:Brown precipitation$$

$$(Mn\left( OH \right)_{2}+{\frac{1}{2}O}_{2}\to Mn{O\left( OH \right)}_{2}\downarrow:Brown precipitation)$$

$$2Mn\left( OH \right)_{3}+2I^{-}+6H^{+}\to2{Mn}^{2+}+I_{2}+{6H}_{2}O$$

$$(Mn{O\left( OH \right)}_{2}+2I^{-}+4H^{+}\to{Mn}^{2+}+I_{2}+3H_{2}O)$$

$$I_{2}+2S_{2}O_{3}^{2-}\to2I^{-}+S_{4}O_{6}^{2-} : iodometric titration$$

**S1 Scheme. Chemical reactions of Winkler’s method**

A FB dispersion was poured into a 100-mL glass biological oxygen demand stoppered bottle (BOD bottle) through siphoning [1]. During this process, 200–300 mL of sample was overflowed to remove the influence of air inside the bottle. Subsequently, 1 mL of fixatives 1 and 2 were added sequentially to the BOD bottle. The fixatives were carefully injected into the bottom of the BOD bottle. The BOD bottle was sealed with a specific lid and inverted 30 times. Brown precipitates were generated depending on the oxygen content. In the case of bubble contamination, we restarted the sample collection. After 10 min, 2 mL of sulfuric acid was added to the surface of the sample liquid to stop the reaction. The BOD bottle was sealed again with a lid and inverted 30 times to dissolve the precipitate under acidic conditions. The sample liquid was transferred to a 200- or 500-mL beaker. After that, titration was applied with a sodium thiosulfate solution using a 10-mL titrator with stirring. When the color became lighter, a starch solution was added for easier identification. The endpoint of the titration was determined using a UV-vis spectrophotometer (V-660; Jasco Corporation, Tokyo, Japan). The titration ended when the 350-nm peak disappeared. The oxygen content of the sample liquid was calculated using Eq. (1):

$oxygen conten \left[ mg/L \right]=\frac{800nv}{V-2r}-0.04$ (1)

where *n* is the concentration of the sodium thiosulfate solution (mol/L), *v* is the titration volume (L), *V* is the volume of the BOD bottle (L) (≈100 mL), and *r* is the injection volume of fixatives (L) (2*r* ≈ 2 mL). In addition, 0.04 mg/L is the correction value from oxygen contamination during the procedure [2].

**References**

1. Saito H, Uchiyama H, Yanaka Y. A Measurement of Oxygen Solubility in Pure Water (Japanese). Japan J water Pollut Res. 1983;6: 237–244. doi:https://doi.org/10.2965/jswe1978.6.237

2. Kitano Y. Dissolved Oxygen Measurement Method for Freshwater and Seawater (Japanese). BUNSEKI KAGAKU. 1964;13: 573–577. doi:https://doi.org/10.2116/bunsekikagaku.13.573
