# Supplementary figures and images for: Development of quantitative and concise measurement method of oxygen in fine bubble dispersion
Source: PLoS One. 2022 Feb 16;17(2):e0264083. doi: 10.1371/journal.pone.0264083 (PMC8849465; doi:10.1371/journal.pone.0264083)

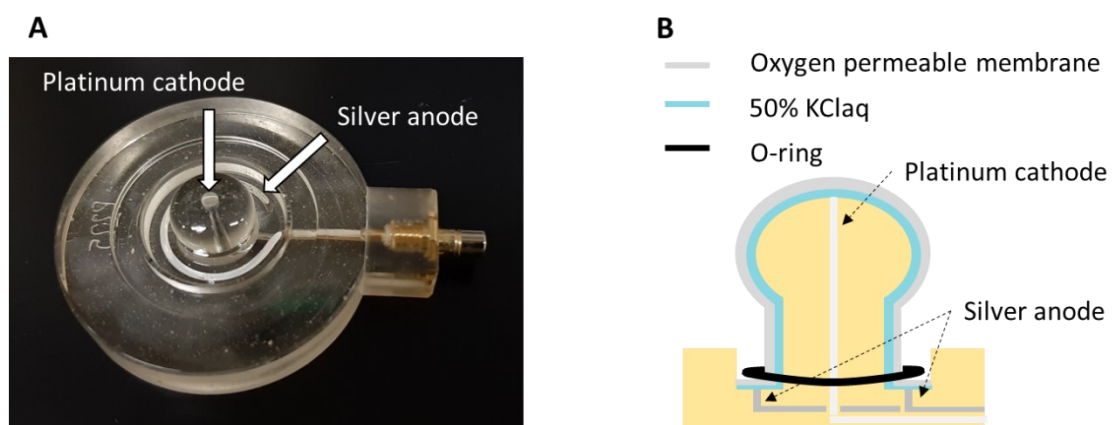

**S1 Fig. Structure of electrode disk.**

Supplement: S1 Fig — (PDF) [file pone.0264083.s003.pdf]
