# Supplementary material for: Development of quantitative and concise measurement method of oxygen in fine bubble dispersion
Source: PLoS One. 2022 Feb 16;17(2):e0264083. doi: 10.1371/journal.pone.0264083 (PMC8849465; doi:10.1371/journal.pone.0264083)
Supplement: S2 Fig — To evaluate the effect of gas-liquid interface on the oxygen level after injecting sample, the screw lid was lifted again (step V) while monitoring the oxygen content in the liquid. The experiments were conducted under two conditions: the gas phase was nitrogen (A to C) and air (D to F). (A and D) Schematic illustration of measurement procedures, which consist of five steps: air saturation (phase I), deoxygenation (phase II), sample injection (phase III), lowering a lid (phase IV), and lifting a lid (phase V). (B and E) Change in oxygen content during a measurement. Discontinuous red lines indicate time point of event: N2 bubbling, sample injection and lowering a lid, and lifting a lid. (C and F) Enlarged view of changes in oxygen content before and after the lifting a lid. (PDF) [file pone.0264083.s004.pdf]

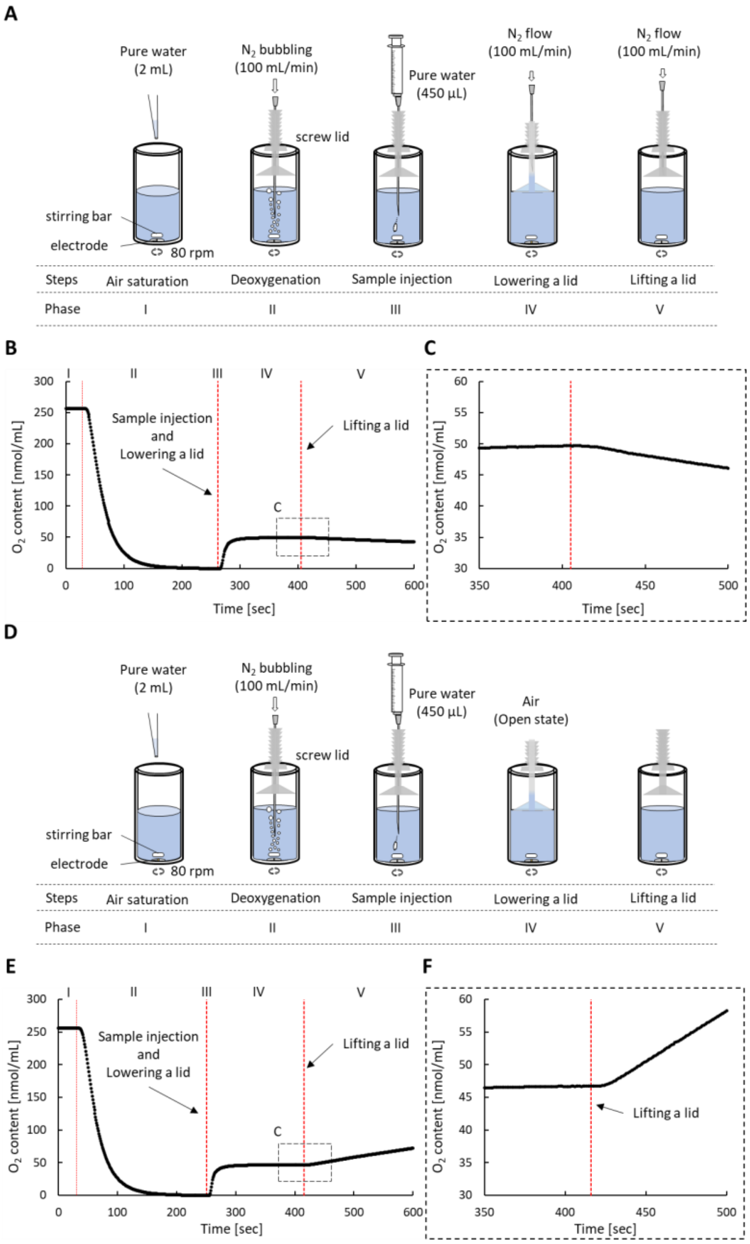


**S2 Fig. Effect of a screw lid on oxygen profile.** To evaluate the effect of gas-liquid interface on the oxygen level after injecting sample, the screw lid was lifted again (step V) while monitoring the oxygen content in the liquid. The experiments were conducted under two conditions: the gas phase was nitrogen (A to C) and air (D to F). (A and D) Schematic illustration of measurement procedures, which consist of five steps: air saturation (phase I), deoxygenation (phase II), sample injection (phase III), lowering a lid (phase IV), and lifting a lid (phase V). (B and E) Change in oxygen content during a measurement. Discontinuous red lines indicate time point of event: $N_2$ bubbling, sample injection and lowering a lid, and lifting a lid. (C and F) Enlarged view of changes in oxygen content before and after the lifting a lid.
