# Supplementary material for: Development of quantitative and concise measurement method of oxygen in fine bubble dispersion
Source: PLoS One. 2022 Feb 16;17(2):e0264083. doi: 10.1371/journal.pone.0264083 (PMC8849465; doi:10.1371/journal.pone.0264083)
Supplement: S3 Fig — Size distribution and concentration of UFBs at A) 20°C, B) 30°C, C) 40°C (n = 3). Approximately 1.0×10^8^ particles/mL were confirmed as same as a manufacturer’s specifications. Size distribution and concentration of MBs at D) 20°C, E) 30°C, F) 40°C (n = 3 at 30°C and 40°C, n = 2 at 20°C). (PDF) [file pone.0264083.s005.pdf]

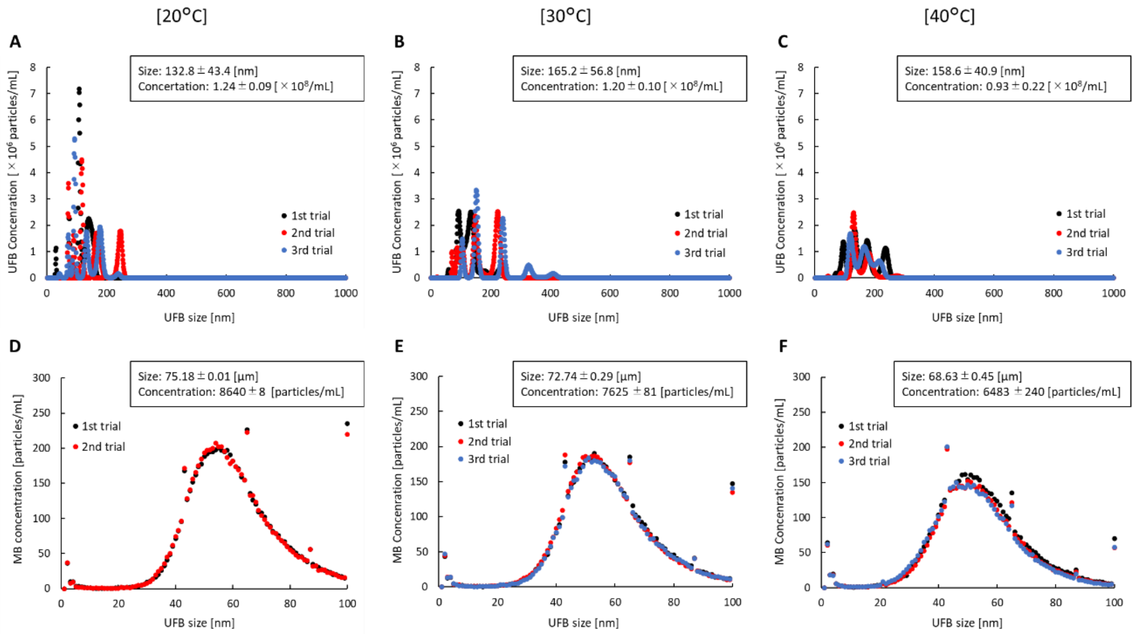

S3 Fig. Properties of fine bubble dispersion. Size distribution and concentration of UFBs at A) 20°C, B) 30°C, C) 40°C (n = 3). Approximately 1.0×10^8 particles/mL were confirmed as same as a manufacturer's specifications. Size distribution and concentration of MBs at D) 20°C, E) 30°C, F) 40°C (n = 3 at 30°C and 40°C, n = 2 at 20°C).
