# Supplementary material for: Development of quantitative and concise measurement method of oxygen in fine bubble dispersion
Source: PLoS One. 2022 Feb 16;17(2):e0264083. doi: 10.1371/journal.pone.0264083 (PMC8849465; doi:10.1371/journal.pone.0264083)
Supplement: S4 Fig — Oxygen content in deoxygenated pure water increased with an increase in the sample volume. There was a strong correlation between the two factors under each temperature condition: r = 0.9994 at 10°C, r = 0.9989 at 20°C, r = 0.9988 at 30°C, and r = 0.9960 at 40°C. Data are presented as the mean ± standard deviation (n = 5). (PDF) [file pone.0264083.s006.pdf]

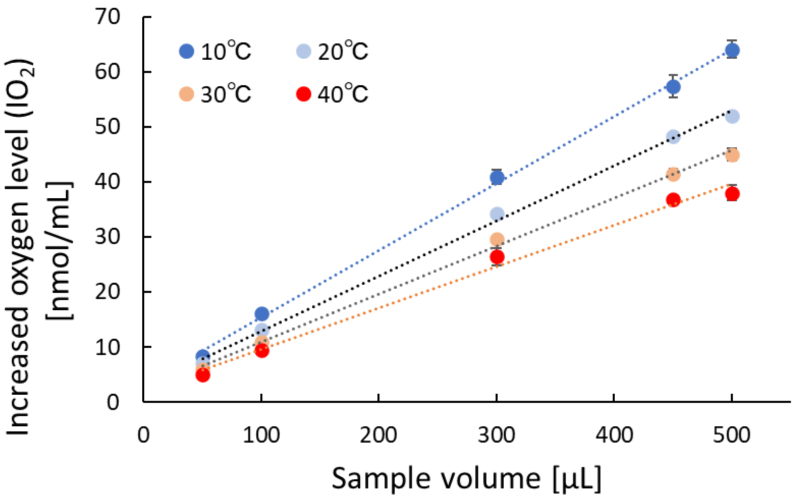

S4 Fig. Relationship between level of oxygen increment and sample volume. Oxygen content in deoxygenated pure water increased with an increase in the sample volume. There was a strong correlation between the two factors under each temperature condition: r = 0.9994 at 10°C, r = 0.9989 at 20°C, r = 0.9988 at 30°C, and r = 0.9960 at 40°C. Data are presented as the mean ± standard deviation (n = 5).
