# Supplementary material for: Development of quantitative and concise measurement method of oxygen in fine bubble dispersion
Source: PLoS One. 2022 Feb 16;17(2):e0264083. doi: 10.1371/journal.pone.0264083 (PMC8849465; doi:10.1371/journal.pone.0264083)
Supplement: S1 Table — Data are presented as the mean ± standard deviation (n = 5). Ave., average; SD., standard deviation; CV., coefficient of variation. (PDF) [file pone.0264083.s007.pdf]

S1 Table. Effects of sample volume and temperature on level of oxygen increment

	10°C		20°C		30°C		40°C	
	Ave. ± SD. [nmol/mL]	CV. [%]	Ave. ± SD. [nmol/mL]	CV. [%]	Ave. ± SD. [nmol/mL]	CV. [%]	Ave. ± SD. [nmol/mL]	CV. [%]
50 μL	8.4 ± 0.5	6.3	7.1 ± 0.4	6.2	5.9 ± 0.1	1.2	5.0 ± 0.3	4.9
100 μL	16.2 ± 0.4	2.4	13.1 ± 0.5	4.0	11.0 ± 0.5	4.1	9.5 ± 0.4	3.9
300 μL	40.9 ± 1.3	3.2	34.3 ± 0.5	1.6	29.7 ± 0.4	1.5	26.4 ± 1.5	5.8
450 μL	57.4 ± 2.0	3.6	48.3 ± 0.7	1.5	41.5 ± 0.9	2.2	36.8 ± 0.5	1.4
500 μL	64.1 ± 1.6	2.5	52.0 ± 0.8	1.5	45.0 ± 1.1	2.4	38.0 ± 1.4	3.6

Data are presented as the mean ± standard deviation (n = 5). Ave., average; SD., standard deviation; CV., coefficient of variation.
