# Supplementary material for: Development of quantitative and concise measurement method of oxygen in fine bubble dispersion
Source: PLoS One. 2022 Feb 16;17(2):e0264083. doi: 10.1371/journal.pone.0264083 (PMC8849465; doi:10.1371/journal.pone.0264083)
Supplement: S2 Table — Data are presented as the mean ± standard deviation (n = 4). Each coefficient of variation includes data under four conditions of sample volume: 100, 300, 450, and 500 μL, excluding 50 μL. Ave., average; SD. standard deviation. (PDF) [file pone.0264083.s008.pdf]

**S2 Table. Coefficient of variation without results of a 50-µL sample volume**

| **Temperature [°C]** | **Coefficient of variation [%]** |
|---|---|
| 10°C | 2.9 ± 0.5 |
| 20°C | 2.1 ± 1.1 |
| 30°C | 2.5 ± 1.0 |
| 40°C | 3.7 ± 1.6 |
| Ave. and SD. | 2.8 ± 0.6 |

Data are presented as the mean ± standard deviation (n = 4). Each coefficient of variation includes data under four conditions of sample volume: 100, 300, 450, and 500 µL, excluding 50 µL. Ave., average; SD. standard deviation.
