# Supplementary material for: Development of quantitative and concise measurement method of oxygen in fine bubble dispersion
Source: PLoS One. 2022 Feb 16;17(2):e0264083. doi: 10.1371/journal.pone.0264083 (PMC8849465; doi:10.1371/journal.pone.0264083)
Supplement: S3 Table — Coefficient of variation in each temperature was calculated from the mean and standard deviation in Fig 4A. Ave., average; SD. standard deviation. (PDF) [file pone.0264083.s009.pdf]

S3 Table. Coefficient of variation for results of FB dispersions

**Temperature [°C]**	**Coefficient of variation [%]**
10°C	1.2
20°C	3.1
30°C	2.9
40°C	3.3
Ave. and SD.	2.6 ± 0.9

Coefficient of variation in each temperature was calculated from the mean and standard deviation in Fig. 4A. Ave., average; SD. standard deviation.
