# Supplementary material for: Development of quantitative and concise measurement method of oxygen in fine bubble dispersion
Source: PLoS One. 2022 Feb 16;17(2):e0264083. doi: 10.1371/journal.pone.0264083 (PMC8849465; doi:10.1371/journal.pone.0264083)
Supplement: S4 Table — Mean oxygen content in fine bubble dispersion at several temperatures measured using the novel method (n = 5), and theoretical DO in oxygen-saturated water calculated form standard DO in air-saturated water. (PDF) [file pone.0264083.s010.pdf]

S4 Table. Oxygen content in FB dispersion and oxygen-saturated water

Temperature [°C]	FB dispersion [mg/L]	Oxygen-saturated water [mg/L]	Difference [%]
15°C	56.82	48.00	18.38
20°C	52.48	43.29	21.24
30°C	44.68	36.00	24.11
40°C	36.94	30.52	21.02

Mean oxygen content in fine bubble dispersion at several temperatures measured using the novel method (n = 5), and theoretical DO in oxygen-saturated water calculated form standard DO in air-saturated water.
